# ARSD, a novel ERα downstream target gene, inhibits proliferation and migration of breast cancer cells via activating Hippo/YAP pathway

**DOI:** 10.1038/s41419-021-04338-8

**Published:** 2021-11-02

**Authors:** Yun Lin, Chun Li, Wei Xiong, Liping Fan, Hongchao Pan, Yaochen Li

**Affiliations:** 1 Central laboratory, Cancer Hospital of Shantou University Medical College, 7 Raoping Road, Shantou, 515041 China; 2 Faculty of Health science, Hull York Medical School, University of Hull, Hull, UK HU6 7RX

**Keywords:** Hormone receptors, Mechanisms of disease

## Abstract

Advanced breast cancer (BC), especially basal like triple-negative BC (TNBC), is a highly malignant tumor without viable treatment option, highlighting the urgent need to seek novel therapeutic targets. Arylsulfatase D (ARSD), localized at Xp22.3, is a female-biased gene due to its escaping from X chromosome inactivation (XCI). Unfortunately, no systematic investigation of ARSD on BC has been reported. In this study, we observed that ARSD expression was positively related to ERα status either in BC cells or tissue specimens, which were associated with good prognosis. Furthermore, we found a set of hormone-responsive lineage-specific transcription factors, FOXA1, GATA3, ERα, directly drove high expression of ARSD through chromatin looping in luminal subtype BC cells. Opposingly, ARSD still subjected to XCI in TNBC cells mediated by Xist, CpG islands methylation, and inhibitory histone modification. Unexpectedly, we also found that ectopic ARSD overexpression could inhibit proliferation and migration of TNBC cells by activating Hippo/YAP pathway, indicating that ARSD may be a molecule brake on ERα signaling pathway, which restricted ERα to be an uncontrolled active status. Combined with other peoples’ researches that Hippo signaling maintained ER expression and ER + BC growth, we believed that there should exist a regulative feedback loop formation among ERα, ARSD, and Hippo/YAP pathway. Collectively, our findings will help filling the knowledge gap about the influence of ARSD on BC and providing evidence that ARSD may serve as a potential marker to predict prognosis and as a therapeutic target.

## Introduction

Breast cancer (BC) has overtaken lung cancer as the most commonly diagnosed cancer and is the most common cause of cancer death among females in 103 countries [1, 2]. Based on the gene expression profiling, the BC has been classified into five intrinsic subtypes with distinct prognostic significance: luminal type A, luminal type B, normal-like, HER-2-positive, and basal-like triple-negative BC (TNBC), in which, 75% of invasive BCs are estrogen receptor 1 + (ESR1 + ) or E2-responsive [3, 4], i.e., luminal subtype. TNBC, accounting for about 15–20% of BCs [5], is more aggressive and has poorer prognosis than other subtypes of BC, owing to lacking receptors to target therapy.

The presence of ERα is considered to be an important marker of slow proliferation, good differentiation, and good prognostic for BC patients who are likely to be responsive to a specific endocrine therapy, such as ER antagonist or aromatase inhibitors [6]. Nevertheless, the vast majority of BC patients cannot escape chemoresistance, eventual recurrence, and metastasis. A more profound understanding for the molecular basis of the genesis and development of BC is still very necessary.

ARSD (Arylsulfatase D) is located at Xp22.3 within a sulfatase gene cluster without any arylsulfatase activity [7, 8]. It is noted that ARSD is one of few female-biased genes and expressed in a stronger female-biased ratio based on *cis* eQTLs analysis [9]. Also, in the analyses of anti-correlated genes and miRNAs, Eric et al. identified 114 female-biased genes in BC, including ARSD gene [10], suggesting that ARSD may be an escaped gene on the X chromosome and tightly related to BC. Unfortunately, no systematic investigation for the effect of ARSD on BC has been reported yet, and it remains unclear whether and how ARSD acts on BC. Thus, it deserves further investigation.

Herein, we provided solid evidences that ARSD, as a novel ERα downstream target gene, inhibits proliferation and migration of breast cancer cells via activating Hippo/YAP pathway. These findings will help filling the knowledge gap about expression and regulation of ARSD gene, as well as the influence of ARSD level on BC.

## Results

### ARSD exhibits higher expression level in normal breast tissue compared to cancer tissue

**Fig. 1:**
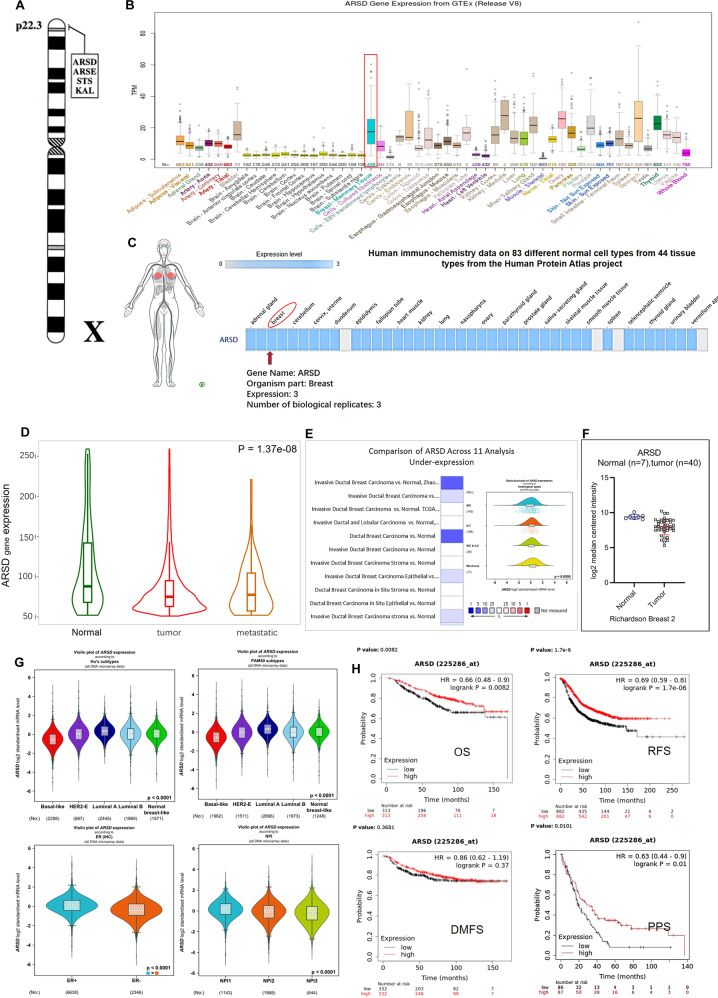
The chromosomal location, expression characteristics of ARSD gene, and its correlation with clinicopathological features in BC. **A** A cluster of sulfatase Genes on Xp22.3. **B** Tissue expression for human ARSD gene. RNA expression distribution of ARSD in 55 tissue types and 6 blood cell types, created by combining the data from three transcriptomics datasets (HPA, GTEx, and FANTOM5) using internal normalization pipeline. **C** ARSD protein expression in breast tissue according to Human immunochemistry data on 83 different normal cell types from 44 tissue types from the Human Protein Atlas project. **D** Comparison of ARSD under-expression across 11 analyses. **E** In Rechardson Breast2 analysis, ARSD presents the lower expression level in breast tumor samples in comparison of normal breast tissues (https://www.oncomine.org/). **F** ARSD presents the lower expression level in breast tumor samples in comparison of normal breast tissues. **G** ARSD presents the lowest expression level in basal-like breast cancer either in Hu’s subtype or in PAM50 subtype. Compared with ER- breast cancers, ARSD presents the higher expression level in ER+ breast cancers. ARSD expression gradually decreases according to the NPI grade. **H** Boxplots created by Kaplan–Meier analysis (https://kmplot.com/analysis/) show the overall survival (OS), relapse-free survival (RFS), distant metastases-free survival (DMFS), and post-progression survival (PPS) of breast cancer patients according to ARSD expression.

As seen in Fig. 1A, ARSD gene is one of the members of a cluster of sulfatase genes mapped to an 8.3 Mb region of Xp22.3. The expression of ARSD transcript and protein was shown in Fig. 1B, C. Obviously, ARSD exhibited high expression level in normal breast tissue relative to other tissues. Meanwhile, a reduced ARSD expression level was observed in breast tumor tissues or metastatic breast tissues when compared with normal breast tissues (*p* = 1.37e-08) (Fig. 1D). Oncomine database shows 26 low expressions of ARSD in 44 BC analyses (Supplementary Fig. 1). Interestingly, in 11 analyses with ARSD under-expression (Fig. 1E), ARSD expression mainly reduced in invasive ductal breast carcinoma compared with other types of BC. Richardson Breast analysis also showed that the expression of ARSD was significantly lower in ductal breast carcinoma than that in their adjacent normal tissues (*p* = 2.13e-7, FC = −2.591) (Fig. 1F). Overall, ARSD exhibits higher expression level in normal breast tissue compared with cancer tissue, which may play a tumor suppression role in BC.

### ARSD gene expression is correlated with ERα status and BC progression

According to bc-GenExMiner v4.5 online tool, we found that luminal A BC presented the highest ARSD expression level, basal-like BC exhibited the lowest expression, and HER2 positive as well as luminal B BC displayed middle expression (*p* < 0.00001; Fig. 1G upper row). Based on ER status, mRNA expression of ARSD was markedly higher in ER-positive than in ER-negative BC (*p* < 0.00001; Fig. 1G lower-left). Additionally, the mRNA expression levels of ARSD were significantly lower in those patients with poor prognosis (NPI2 and NPI3) when compared to patients with good prognosis (NPI1) (*p* < 0.00001; Fig. 1G lower-right). KM plotter analysis showed a significant correlation between high ARSD expression level and the favorable overall survival (OS) (with the HR of 0.66, *p* = 0.0082), relapse-free survival (RFS) (HR of 0.69, *p* = 1.7-e6), and progression-free survival (PFS) (HR of 0.63, *p* = 0.01) in BC patients (Fig. 1H).

Furthermore, we analyzed the correlations between ARSD expression and the clinical and pathological characteristics of BC patients by IHC. The results of statistical analyses were shown in Table 1. The positive expression rate of ARSD in the BC tissues from 102 cases were 54.90% (56/102). The expression of ARSD was significantly associated with molecular subtype (*p* < 0.001), clinical stage (*p* = 0.004), histological stage (*p* < 0.001), ER (*p* < 0.001), PR (*p* = 0.004), Ki67 (*p* = 0.003), and TopIIα (*p* = 0.013), respectively, but it was in no correlation with age, tumor size, LN metastasis, Her2, p53, VEGF. These results were further supported by online database (Supplementary Fig. 2A). Notably, ARSD positive ratio in luminal subtype BC reached 77.97% (46/59), whereas the positive ratio was only 23.26% (10/43) in basal-like subtype BC. Similarly, 78.57% (44/56) of patients with ARSD positive expression were ER+, while only 21.43% (12/56) were ER- cases. Collectively, these data suggest that ARSD expression highly depends on ER status, which should be used as a prognosis marker.

**Table 1:** Demographic and clinicopathologic characteristics of breast cancer patients.

Variable	Cases	Percent (%)	ARSD		*P* value ^a^
			Negative	Positive	
Age (years)					
≤50	43	42.16	20	23	0.807
>50	59	57.84	26	33	
Tumor size (cm)					
≤3	53	51.96	22	31	0.449
>3	49	48.04	24	25	
LN metastasis					
Yes	54	52.94	22	32	0.348
No	48	47.06	24	24	
Molecular subtype					
Luminal	59	57.84	13	46	**<0.001**
Basal-like	43	42.16	33	10	
Clinical stage					
I	23	22.55	5	18	**0.004**
II	41	40.20	16	25	
III/IV	38	37.25	25	13	
Histological stage					
I	37	36.27	8	29	**<0.001**
II	26	25.49	11	15	
III	39	38.24	27	12	
ER					
Negative	44	43.14	32	12	**<0.001**
Positive	58	56.86	14	44	
PR					
Negative	62	60.78	35	27	**0.004**
Positive	40	39.22	11	29	
Her2					
Negative	50	49.02	19	31	0.158
Positive	52	50.98	27	25	
Ki67					
Low	43	42.16	12	31	**0.003**
High	59	57.84	34	25	
VEGF					
Negative	41	40.20	20	23	0.807
Positive	61	59.80	26	33	
p53					
Negative	51	50.00	22	29	0.691
Positive	51	50.00	24	27	
Top IIα					
Low	47	46.08	15	32	**0.013**
High	55	53.92	31	24	

^a^Chi-square test.

### ARSD presents high expression in luminal subtype BC cells but low expression in TNBC cells

**Fig. 2:**
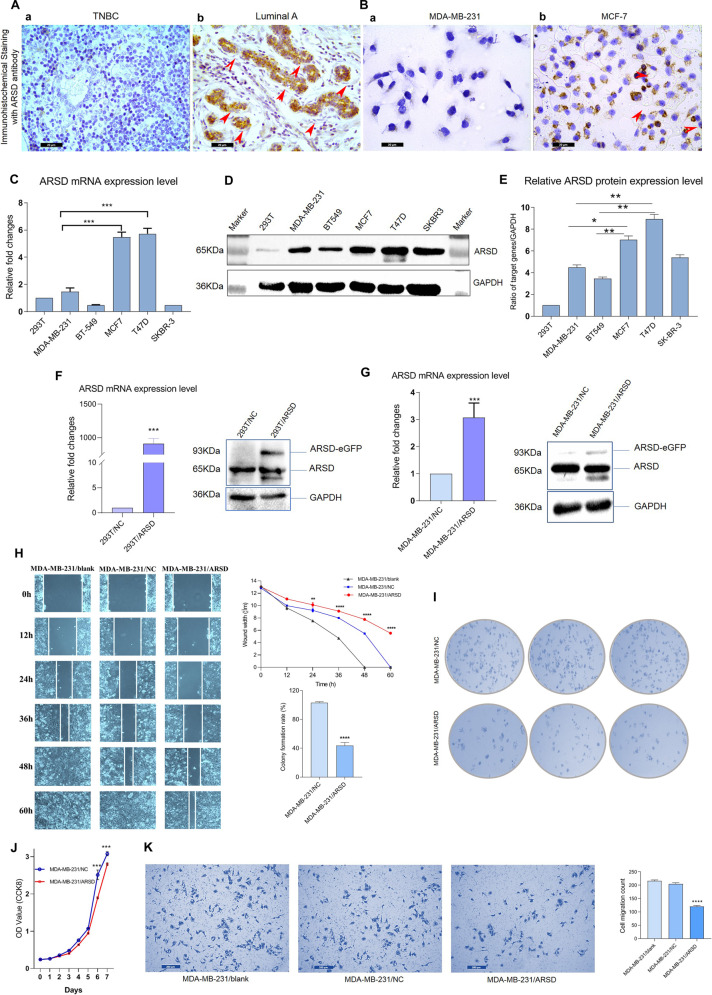
ARSD exhibits high expression in luminal subtype BC cells or specimens, and ARSD overexpression has inhibitory effects on the proliferation and migration of BC cells. **A** Immunohistochemistry staining of BC tissues, including basal like and luminal subtype BC tissues. The positive signals are marked with red arrow heads. **B** Immunocytochemistry staining of BC cells, such as MCF-7 and MDA-MB-231 cells, with ARSD specific antibody. **C** The relative expression of ARSD mRNA is detected by qRT-PCR in HEK293T and five breast cancer cell lines. **D** ARSD protein expression is detected by Western blotting in HEK293T and five breast cancer cell lines. The ARSD expression is represented by a band of 65 KDa corresponding to ARSD protein full length. GAPDH is shown as a loading control of samples. **E** The gray scale analysis is performed using a gel analyzer. Boxplot shows that the relative content of the target protein is the ratio of the target protein to the gray value of corresponding internal reference bands. **F** The overexpression efficiency of ARSD was tested in HEK293T and **G** in MDA-MB-231 cells by using RT-PCR and Western blotting. **H** Wound healing assay of MDA-MB-231/NC cells and MDA-MB-231/ARSD cells. **I** Colony formation of MDA-MB-231/NC cells and MDA-MB-231/ARSD cells on a plastic substrate. The colony formation rate was quantified using ImageJ software 14 days after plating. **J** Cell proliferation analysis of negative control (MDA-MB-231/NC cells) and MDA-MB-231/ARSD cells is detected every 24 h after plating (day 0) by using CCK-8 assay. **K** Transwell assay of MDA-MB-231/NC cells and MDA-MB-231/ARSD cells. Error bars are ±SEM (*p < 0.05, **p < 0.01, ***p < 0.001).

We also carried out immunocytochemical staining against ARSD in BC tissues and cell lines. Obviously, strong positive expression of ARSD was observed in luminal subtype BC tissues, while it was hard to find positive signals in TNBC tissues (Fig. 2A). Similarly, no positive signal was found in MDA-MB-231 cells (Fig. 2B-a), whereas robust ARSD immunoreactivity was observed uniformly in the cytoplasm of MCF-7 cells (Fig. 2B-b). The qRT-PCR and Western blotting in five human BC cell lines including MCF-7, T47D, SKBR3, MDA-MB-231, and BT549 (HEK293T cells were also detected as a reference) uncovered that ARSD expression was significantly different among the five BC cell lines. In detail, those highly invasive BC cell lines, such as MDA-MB-231 and BT-549 presented lower expression levels of ARSD, whereas the ER positive MCF-7 and T47D cell lines presented higher expression levels of ARSD (Fig. 2C–E and Supplement Fig. 2B). The expression level of ARSD in SKBR3 cells was moderate. Collectively, these data indicate that ARSD expression is increased in luminal subtype BC cells, and decreased in TNBC or HER2 + BC cells that are associated with highly invasive behavior and poor prognosis.

### Ectopic overexpression of ARSD inhibits the proliferation, colonies formation, and migration of BC cells, maintaining a less aggressive phenotype in BC cells

In order to explore the effect of ARSD on the biological functions of BC cells, ARSD overexpression vector was constructed and verified (Fig. 2F, G and Supplement Fig. 2C). ARSD overexpression markedly inhibited the migration (Fig. 2H, Supplementary Figs. 3 and 4A) and the colony formation of TNBC cell (Fig. 2I and Supplementary Fig. 4B). The proliferation of MDA-MB-231 and BT-549 was significantly suppressed in ARSD overexpression group compared to the control cells (Fig. 2J and Supplementary Fig. 4C). Furthermore, transwell assays showed that the invasion ability of TNBC cells was markedly inhibited by ARSD overexpression (Fig. 2K and Supplementary Fig. 4D). By contrary, once ARSD was knocked down, the capacities of proliferation and migration of MCF-7 cells were significantly increased (Supplementary Fig. 4E–H). Taken together, these data suggest that the ARSD expression in breast tissue may protect from women to cancer.

### High Xist expression, DNA methylation, and repressive histone modifications implicate ARSD inactivation in MDA-MB-231 cells

**Fig. 3:**
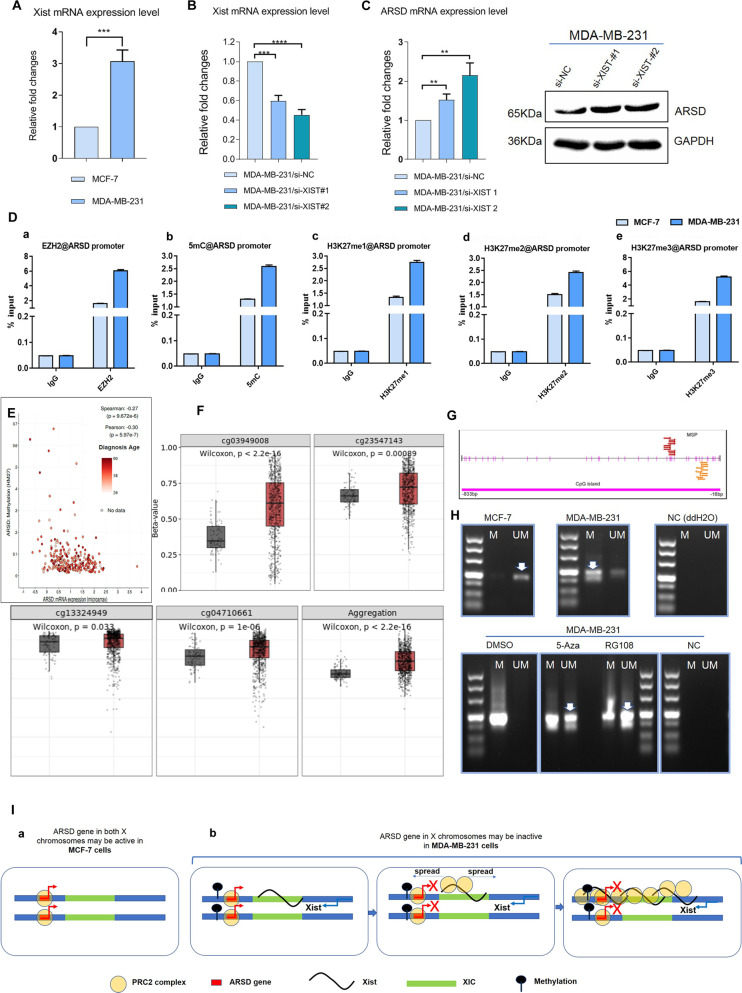
ARSD exhibits lower expression in MDA-MB-231 cells due to that it is still subjected to XCI. **A** Xist expression levels of MCF-7 and MDA-MB-231 cells are tested by qRT-PCR. **B** Validation of Xist knockdown efficiency by qRT-PCR. **C** ARSD mRNA and protein expression levels are tested after Xist knockdown. **D** ARSD promoter occupancy in MCF-7 and MDA-MB-231 cells is detected using ChIP-qPCR with **D-a** EZH2, **D-b** 5-mC, **D-c** H3K27me1, **D-d** H3K27me2, and **D-e** H3K27me3 specific antibodies, respectively. Light blue and dark blue bars indicate loci in MCF-7 and MDA-MB-231 cells, respectively. Experiments were duplicated and two data sets are concordant (R2 = 0.92). The ratios of enrichment of EZH2, 5mC, H3K27me, H3K27me2, and H3K27me3 were higher in MDA-MB-231 cells than that in MCF-7 cells. Error bars are ±SEM (*p < 0.05, **p < 0.01, ***p < 0.001). **E** A negative correlation exists between ARSD mRNA and ARSD methylation (HM27). **F** The probes Cg3949008, Cg 23547143, Cg13324949, and Cg04710661 were used to evaluate and compare the methylation levels of ARSD gene promoter between normal and tumor tissue (http://www.bioinfo-zs.com/smartapp/). **G** ARSD promoter is analyzed by using Methyl Primer Express Software v. 1.0. **H** MSP analysis of the methylation status of the ARSD promoter. “U” indicates unmethylated amplification, and “M” indicates methylated amplification. Lower panel shows MDA-MB-231 cells were, respectively, treated with 5-Aza or RG108 for 5 days prior to DNA isolation. **I** Synopsis of ARSD gene subjected to XCI in MDA-MB-231 cells instead of that in MCF-7 cells. Data are presented as the means ± SD of three independent experiments. *p < 0.05, **p < 0.01 and ***p < 0.001 (Student’s *t*-test) as compared to control cells.

Given that ARSD is an escaped gene on the X chromosome [11], the expression of lncRNA Xist was therefore examined in MCF-7 and MDA-MB-231 cells by qRT-PCR. The quantitative analysis uncovered that the expression level of lncRNA Xist in MDA-MB-231 cells was higher than that in MCF-7 cells (Fig. 3A). Followed by knockdown of Xist with siRNAs, the expression of Xist was markedly downregulated (Fig. 3B), resulting in raised ARSD mRNA as well as protein expression in MDA-MB-231 cells (Fig. 3C). Besides Xist RNA coating, maintenance of X-inactivation is achieved through a combination of different repressive mechanisms, including polycomb 2 group protein recruitment, repressive histone modifications, and DNA methylation. ChIP-qPCR revealed increased 5-mC occupancy at the promoter region of ARSD in MDA-MB-231 cells when compared with that in MCF-7 cells, suggesting the results that the CpG island in the promoter region of ARSD was hypermethylated in MDA-MB-231 cells. In addition, the occupancies of EZH2, H3K27me1, H3K27me2, and H3K27me3 were also significantly raised in ARSD promoter in MDA-MB-231 cells (Fig. 3D). Furthermore, based on cBioportal database, there is high negative correlation between ARSD mRNA expression levels and the methylation states of ARSD promoter region in BC (Fig. 3E). Also, the average beta-values were aggregately higher in tumor tissue than that in the normal tissue (Fig. 3F). Based on Methyl Primer Express v1.0 software, a large CpG island that locates −833bp~−16bp from the transcription start site was found (Fig. 3G). Next, specific PCR (MSP) was conducted. The results showed that the CpG island of ARSD gene promoter was completely in unmethylation status in luminal A subtype MCF-7 cells, whereas hypermethylated status were observed in MDA-MB-231 cells (Fig. 3H upper). Notably, methylated DNA were converted into unmethylation states after 5-Aza or RG108 treatment, whereas no change of DNA methylation status was observed in those MDA-MB-231 cells treated with DMSO (Fig. 3H lower). Collectively, these data indicate that Xist RNA coating, DNA methylation, and repressive histone modifications play significant roles in ARSD gene expression, which may cause ARSD fail to escape from XCI in MDA-MB-231 cells (Fig. 3I).

### ARSD is directly regulated by luminal subtype transcription factors, FOXA1, GATA3, and ERα

**Fig. 4:**
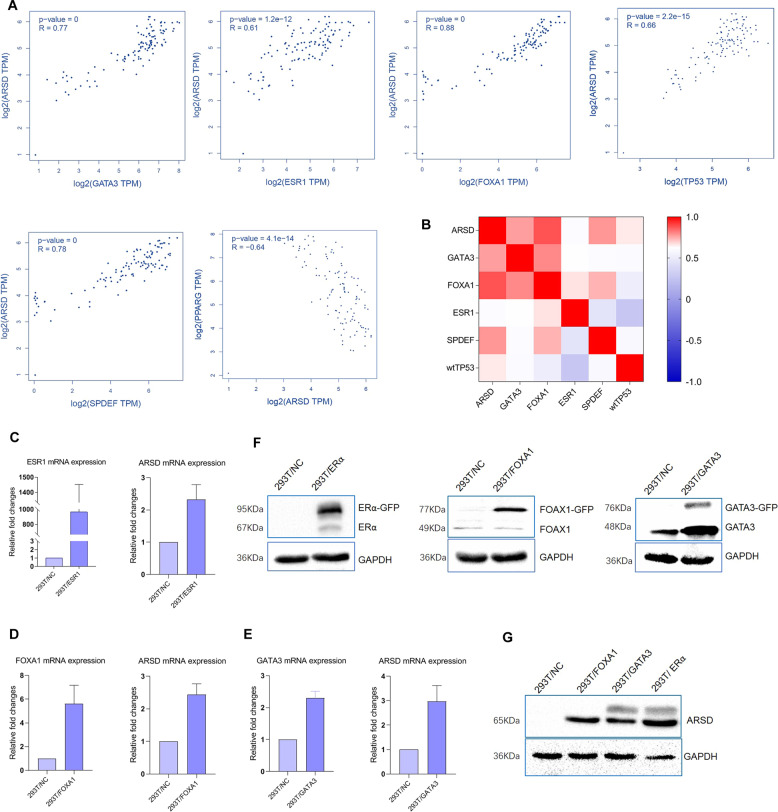
A positive correlation exists between ARSD and FOXA1, GATA3 or ESR1, respectively. **A**–**C** qRT-PCR results reveal that ectopically overexpressing FOXA1, GATA3, and ESR1 significantly upregulate ARSD expression level, respectively. **D**, **E** Western blotting results reveal that ectopically overexpressing FOXA1, GATA3, and ESR1 significantly upregulates ARSD protein level, respectively. **F** Pearson correlation between the gene expression of ARSD and luminal subtype transcriptional factors, e.g., FOXA1, GATA3, ESR1, SPDEF, wtTP53, and PPARG in breast cancer. Data was adopted from the Gene Expression Profiling Interactive Analysis (GEPIA) resource. **G** Visualizing the correlation matrix as a heatmap.

**Fig. 5:**
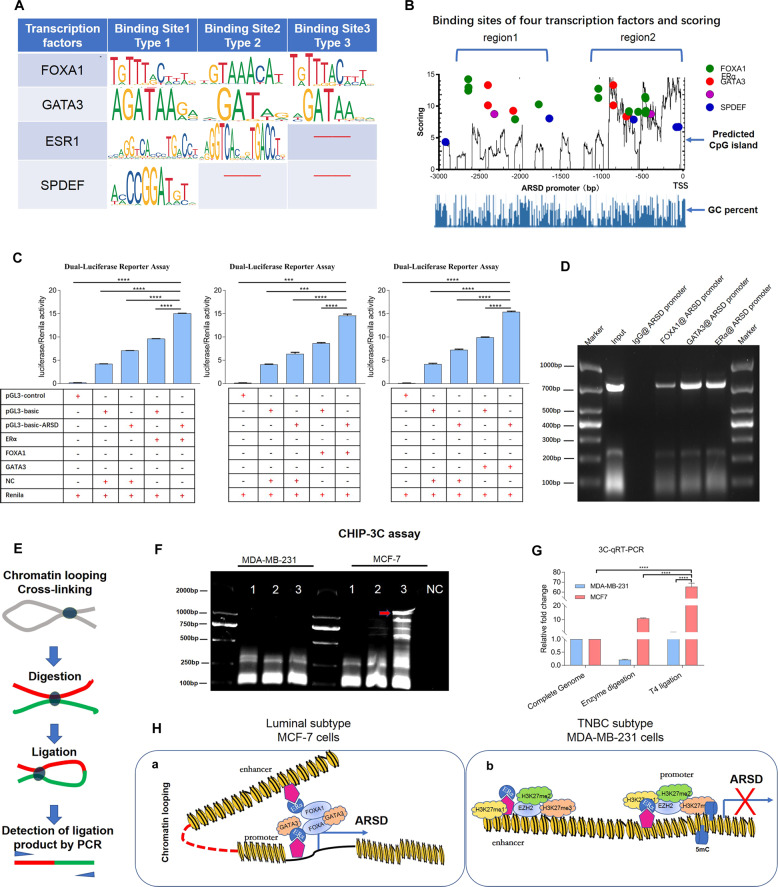
ARSD is regulated by FOXA1/GATA3/ ERα mediated chromatin looping at the transcriptional level. **A** The predicted binding sites of FOXA1, GATA3, and ESR1 in the ARSD enhancer/promoter by Jaspar online software. **B** The pattern diagram shows the scores, CpG islands, and the corresponding locations of FOXA1, GATA3, and ERα binding site in ARSD enhancer/promoter. **C** The fragment of ARSD enhancer/promoter containing FOXA1, GATA3, and ESR1 binding site (−2786bp~−2050bp) was inserted into the luciferase reporter vectors by two restricted endonuclease (Sac I (CGAGCTCG) and Sma I (TCCCCCGGGGGA)). HEK293T-FOXA1, GATA3 or ERα overexpression cells and HEK293T-NC control cells were co-transfected pGL3-enhancer ARSD-promoter reporter vector, pGL3-enhancer vector or pGL3-control vector with Renilla luciferase reporter vector, respectively. Luciferase activity was normalized to Renilla. *p < 0.05, **p < 0.01 and ***p < 0.001 (Student’s *t*-test) as compared to control cells. Data are presented as mean ± SD (*n* = 3). **D** The ChIP-PCR assay used normal IgG (IgG) or anti-FOXA1, GATA3 or ERα antibodies to determine whether FOXA1, GATA3 or ERα can bind the corresponding binding site in the ARSD enhancer/promoter in MCF-7 cells. Input was used to be as positive control, and IgG was used to be as negative control. **E** The experimental strategies of chromosome conformation capture (3 C). This assay was applied to determine higher-order chromatinic interactions at the ARSD enhancer/promoter locus upon activation in MCF-7 cells. **F** The PCR is performed with 3 C template and primers. Analysis of 3 C data by gel electrophoresis. Verification that premixing primers from the ARSD enhancer/promoter locus. **G** qRT-PCR with 3 C template and primers. **H** Schematic illustration of the ARSD expression regulated by GATA3/FOXA1/ERα network via mediating chromatin looping. **H-a** The chromatin loop formation mediated by the pioneer factors, GATA3 and FOXA1, which open the compressed chromatin conformation and bind to the ERα/ERE complex to enhance the ARSD expression in MCF7 cells. **H-b** Epigenetic modification of ARSD promoter/enhancer in MDA-MB-231 cells by DNA methylation and EZH2, an enzymatic catalytic subunit of polycomb repressive complex 2 (PRC2) that can alter downstream target genes expression by trimethylation of Lys-27 in histone 3 (H3K27me3).

Considering that FOXA1, GATA3, and ERα are important luminal subtype transcription factors (Supplementary Fig. 5), FOXA1, GATA3, and ERα overexpression vectors were constructed. The results showed about 3-fold upregulation of ARSD expression along with the FOXA1, GATA3 or ERα transfection (Fig. 4A–C). The Western blot assay also confirmed that FOXA1, GATA3 or ERα overexpression led to a robustly raised expression of ARSD (Fig. 4D, E and Supplement Fig. 6). These results were also supported by the co-expression analyses using GEPIA database (Fig. 4F, G). Analysis of the sequence of promoter and first non-encoding exon of ARSD (−3000bp~+64 bp) revealed that there were dense clusters of FOXA1 and GATA3 binding sites, which were around two canonical ERα binding sites Fig. 5A, B. The dual luciferase reporter assay showed that ARSD promoter activity was significantly increased, once ERα, FOXA1, and GATA3 were overexpressed, respectively, suggesting that ERα, FOXA1, and GATA3 could effectively elicit ARSD expression (Fig. 5C; *p* < 0.05). As expected, chromatin immunoprecipitation (ChIP) assay confirmed these bindings along with ERα, FOXA1, and GATA3 antibodies pull-down (Fig. 5D). To sum up, these results demonstrate that ERα, FOXA1, and GATA3 directly activate ARSD expression at transcriptional level.

### FOXA1, GATA3, and ERα enhance ARSD expression via chromatin looping in MCF-7 cells

To determine whether FOXA1, GATA3, and ERα enhanced the expression of ARSD gene via chromosome conformation 3D in MCF-7 cells and whether the chromatin loop was compromised in MDA-MB-231 cells, chromosome conformation capture (3 C) assay was conducted with specific 3 C primers (Fig. 5E and Supplementary Fig. 7), which can be used to analyze the overall spatial organization of chromosomes [12]. The results showed that the PCR product with expected size about 1500 bp was only obtained in MCF-7 cells, suggesting that chromatin loop only formed in MCF-7 cells but not in MDA-MB-231 cells (Fig. 5F, G). Overall, these data indicate that FOXA1, GATA3, and ERα are upstream molecules of ARSD via chromatin looping in luminal subtype BC cells (Fig. 5H).

### Ectopic ARSD overexpression activate Hippo/YAP pathway in BC cells

**Fig. 6:**
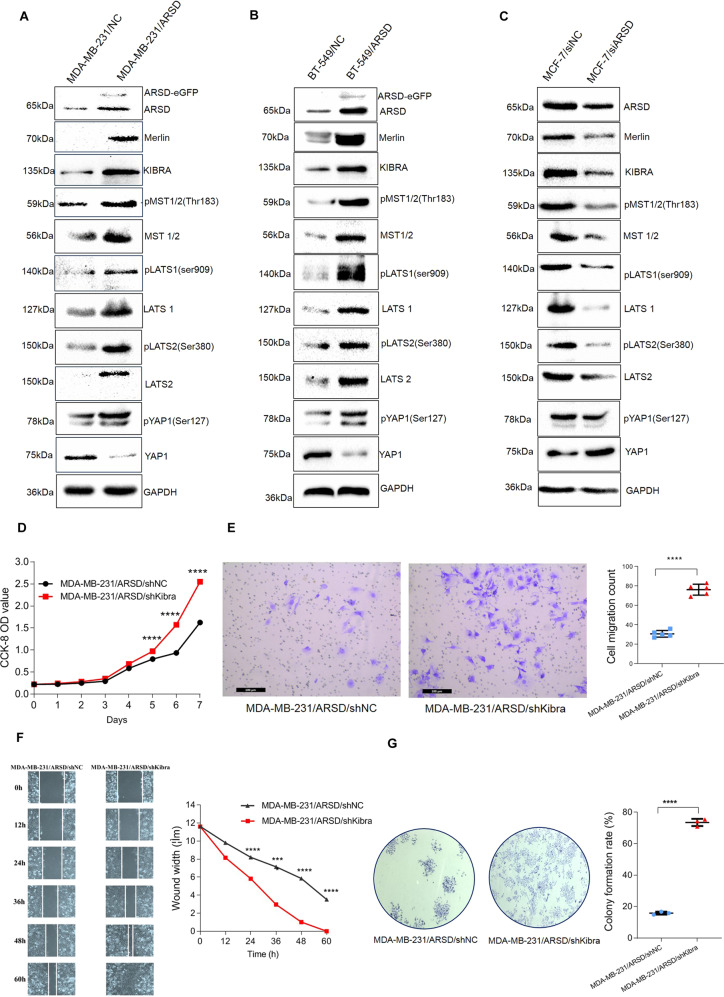
Ectopic ARSD overexpression activates Hippo/YAP pathway in breast cancer cells. **A**, **B** Western blotting verifies that ARSD overexpression activates Hippo/YAP pathway in MDA-MB-231 and BT-549 cells. **C** ARSD knocking down inactivates Hippo/YAP pathway in MCF-7 cells. All molecular weights are in KDa. All experiments were repeated three times independently, yielding similar results, representative images are shown. **D** Cell proliferation analysis of negative control (MDA-MB-231/ARSD/shNC cells) and MDA-MB-231/ARSD/shKibra cells is detected every 24 h after plating (day 0) by using CCK-8 assay. **E** Transwell assay of MDA-MB-231/ARSD/shNC cells and MDA-MB-231/ARSD/shKibra cells. **F** Wound healing assay of MDA-MB-231/ARSD/shNC cells and MDA-MB-231/ARSD/shKibra cells. **G** Colony formation of MDA-MB-231/ARSD/shNC cells and MDA-MB-231/ARSD/shKibra cells on a plastic substrate. The colony formation rate was quantified using ImageJ software 14 days after plating; error bars are ±SEM (*p < 0.05, **p < 0.01, ***p < 0.001).

Recently, it has been reported that Hippo effector YAP is a key regulator of cell–matrix interaction [13]. Considering that ARSD should play crucial role in regulating ECM remolding, we therefore examined whether there is an interaction relationship between ARSD and Hippo/YAP pathway. As shown in Fig. 6A–C, the expression levels of the upstream molecules and core kinase cassette proteins, such as Kibra (WWC1), Merlin/NF2, pLATS1 (Ser909)/pLATS2 (Ser380), pMST1/2 (Thr183) as well as pYAP (Ser127) were upregulated or downregulated at protein levels accompanied by overexpressing or knocking down ARSD in MDA-MB-231 or MCF-7 BC cells, respectively. Interestingly, the total protein levels of LATS1/LAST2, MST1/2 were also found to upregulate or downregulate along with overexpressing or knocking down ARSD. Simultaneously, a robust increase or reduction of the phosphorylated YAP1(Ser27) expression was observed to associate with ARSD overexpression or knocking down. By contrast, the total protein level of core effector YAP reduced or increased accompanied by overexpressing or knocking down ARSD. These results were further supported by GEPIA online database (Supplementary Fig. 8). To further confirm that ARSD plays a role through Hippo/YAP pathway, the rescue functional experiments were conducted by knocking down Kibra in MDA-MB-231 cells with ARSD overexpression background. As seen in Fig. 6D–G, ARSD overexpression combined Kibra knocking down partially recovered the abilities of proliferation and migration of MDA-MB-231 cells compared with those cells with ARSD overexpression alone. In summary, these results suggest that overexpressing ARSD can activate the Hippo/YAP pathway in BC cells.

## Discussion

ARSD gene is unique in many aspects. First, it is one of the members of a cluster of sulfatase genes (ARSC, ARSD, ARSE, ARSF, ARSH) mapped to an 8.3 Mb region of Xp22.3 [8]. In this region, almost all genes escape XCI and express in a variety of normal tissues. Second, the expression level of ARSD may be dynamic, which depends on XCI or reactivation (escape inactivation) regulated by epigenetic regulation [14, 15]. Third, unlike most of the X-linked genes that are subject to XCI, ARSD escapes from XCI and shows a female-expression bias in the majority of tissues including breast tissue. Many of the genes in this category are known oncogenes or tumor suppressors (e.g., DDX3X, TRAPPC2, and TCEANC), they play crucial roles in women’s cancer [16]. Nevertheless, there are very few studies about ARSD gene. Just recently, ARSD has been demonstrated to participate in the post-translational changes of some proteins that possess a critical role in cancer progression [17]. Unfortunately, whether and how ARSD impacts on BC are still unknown. What is the basic function of ARSD gene?

In the present study, we conducted a comprehensive study on ARSD gene and found that luminal subtype BC possessed significantly higher ARSD expression than TNBC, and that ARSD expression was highly correlated with clinical outcomes in BC patients. We also found ARSD expression highly depended on luminal-subtype transcription factors, such as FOXA1, GATA3, and ERα, either in BC cells or in BC specimens. Besides, ARSD overexpression can inhibit the proliferation and migration of TNBC cells by activating Hippo/YAP pathway. We thereafter speculated that ARSD may be as a novel tumor suppressor in BC.

It is worth to think why ARSD expression is significantly downregulated in TNBC. Considering its X chromosome location, we guessed that there might be at least two main mechanisms leading to the downregulation of ARSD expression in TNBC.

Firstly, the decreased expression of ARSD gene in TNBC cells may be due to the possibility that ARSD gene is still subjected to XCI or failed in escaping XCI. To our knowledge, XCI is a dosage compensation mechanism in females that results in the inactivation of one of two X chromosomes in females [18]. Notably, a small number of genes still escape from XCI and express apparently at different level although various means collaborate to ensure inactivation, such as Xist coating, DNA methylation, and inhibitory epigenetic modification etc.

Xist is a 17-kb long non-coding RNA (lncRNA) encoded by Xist gene. Usually, XCI is initiated through coating of the nascent inactive X chromosome by Xist. After that, Xist is required for long-term maintenance of random XCI. In addition, XCI is accompanied by CpG islands methylation and inhibitory histone modification [19, 20]. Typically, the Xist lncRNAs covering X chromosome attract PRC2 (polycomb repressive complex 2) and PRC1 and directly interact with EZH2, the catalytic subunit of PRC2, both in vivo and in vitro. EZH2 catalyzes H3K27 trimethylation (H3K27me3) and inhibits H3K27 acetylation (H3K27ac) in order to silence the X-chromosome [21].

A series of epigenetic events above mentioned were corroborated by us in MDA-MB-231 cells, e.g., (1) Higher expression level of Xist was observed; once Xist was knocked down, the expression of ARSD was raised. (2) More methylated PCR products were obtained, and 5-Aza treatment reactivated ARSD expression. Simultaneously, more 5-mC were enriched to the enhancer/promoter. (3) More EZH2, H3K27me1, H3K27me2, and H3K27me3 occupied the enhancer/promoter of ARSD. These evidences demonstrated that ARSD gene was still subjected to XCI or re-silenced in MDA-MB-231 cells. We therefore propose that XCI state may dynamically change between molecular subtypes of BC in the process of tissue and organ development [22].

Secondly, the decreased ARSD expression in TNBC cells may be due to that ARSD gene is an ERα downstream target gene. In this study, we verified that ARSD was directly regulated by ERα through correlation analysis, overexpressing/knocking down experiments, predicting TFs binding site, ChIP, and luciferase assay. Specifically, both FOXA1 (forkhead box A1) and GATA3 (GATA-binding protein 3, a zinc finger transcription factor) involved in the regulation of ERα on ARSD. Overwhelming evidence shows that FOXA1, GATA3, and ERα are essential for luminal subtype-specific gene regulation and molecular subtype switching [23]. It is known that ERα drives the differentiation of luminal cells [24], and FOXA1, as a pioneering factor, prepares genomic sites for ERα to bind with chromatin [25]. GATA3, a lineage-restricted transcription factor, is essential for the mammary-gland morphogenesis and luminal-cell differentiation [26], which is also considered as an ESR1-cooperating transcription factor and the upstream of FOXA1 in mediating ESR1 binding by shaping enhancer accessibility [27]. Consequently, we deduced that ARSD may be a novel downstream target gene of ERα, and three TFs, FOXA1, GATA3, and ERα, may form a functional enhanceosome to drive the transcription of ARSD in MCF-7 BC cell.

It is well known that ERα is a pivotal molecule to induce long-distance chromatin interactions with ERα binding site and transcription start site (TSS) through chromatin looping [28]. Via chromatin looping, the enhancers region may serve as transcription factor depots for regional TSSs [29]. This theory prompted us to further investigate whether chromatin loop was formed at the enhancer/promoter of ARSD gene in MCF-7 cells. Unsurprisingly, through chromatin conformation capture (3 C) technique, chromatin loop formation was observed in MCF-7 cells rather than in MDA-MB-231 cells. There may be multiple reasons resulting in the failure of chromatin loop formation in MDA-MB-231 cells: (1) TNBC cells were absent in ERα expression. (2) The expression of FOXA1 and GATA3 at the protein level was below the limit of detection, which were the important elements mediating the chromatin loop formation. (3) The abundant of EZH2, 5-mC and H3K27me3 were enriched in the enhancer/promoter of ARSD, which may arrest the chromatin loop formation. In stark contrast, the co-occupied sites by ERα, FOXA1, and GATA3 are associated with highest p300 co-activator recruitment, RNA Pol II occupancy, and chromatin opening [30]. High-concentration depots of co-activator multi-protein complexes could drive this prodigious activity of ARSD gene expression. Therefore, it is easy to understand why ARSD presents higher expression in MCF-7 cells and lower in MDA-MB-231 cells.

**Fig. 7:**
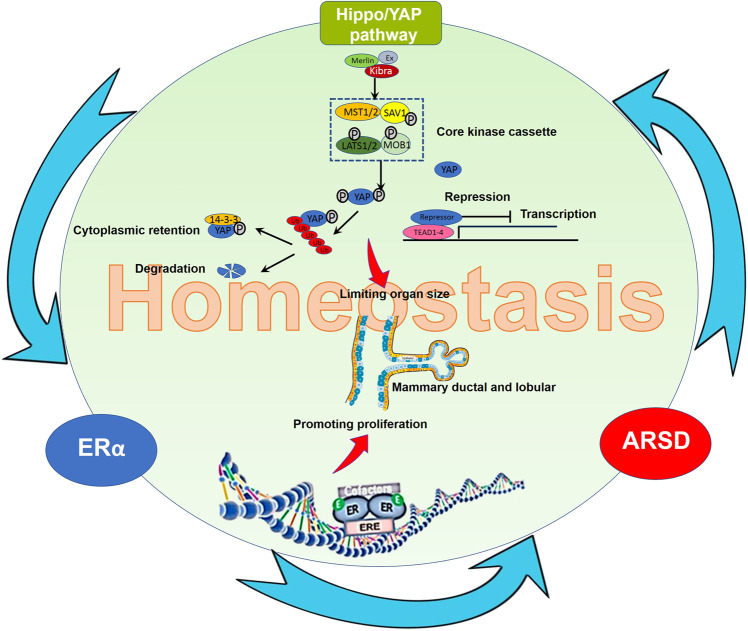
There might exist a constrained workflow model among ERα, ARSD, and Hippo/YAP pathway. ARSD, as a molecule brake on ERα signaling pathway, restricts ERα in an uncontrolled active state and avoids the overgrowth caused by ERα through activating Hippo/YAP pathway in order to reach homeostasis in breast luminal epithelial cells.

ERα has long been known to play a crucial role in breast epithelial cell proliferation and survival, as well as mammary tumorigenesis mediated by its genomic and non-genomic actions [31]. Nevertheless, as the target gene of ERα, why can enforced ARSD expression significantly suppress BC cells proliferation and migration? In present study, we found that accompanying by ARSD overexpression or knockdown, Hippo/YAP pathway was significantly turned on or turned off. Activation of the Hippo pathway is converged to its main effector YAP, whose phosphorylation leads to the cytoplasmic retention and protein degradation [32]. When the Hippo signaling is turned off, the unphosphorylated YAP is translocated from cytoplasm into nucleus and interacts with transcription factors TEAD1–4, then promoting downstream genes that are involved in cell survival and cell growth [33]. Recently, the available data suggest that Hippo/YAP pathway has been considered as a brake on cell division that can prevent organs from growing larger, once they have reached the appropriate size [34, 35]. Giancotti et al. reported that LATS1/2 could facilitate ERα ubiquitylation by the E3 ligase CRL4^DCAF1^ [36]. More recently, Adrian et al. and Guan et al., respectively, identified that there was a direct interaction between Hippo and ERα signaling [37, 38], in which Hippo signaling maintained ER expression and regulated ER+ BC growth. Accordingly, we proposed that there might exist a constrained workflow model among ERα, ARSD, and Hippo/YAP pathway (Fig. 7), in which, ARSD may be a molecule brake on ERα signaling pathway, which restricts ERα in an uncontrolled active state and avoids the overgrowth caused by ERα through activating Hippo/YAP pathway in order to reach homeostasis in breast luminal epithelial cells. Although previous studies have shown that mammalian sulfatase enzymes participate in various processes, such as hormone regulation, lysosomal degradation, and modulation of several signaling pathways [39, 40], the studies of ARSD on Hippo/YAP pathway have not been reported yet.

It is also worth noting that, in the present experiment, besides the phosphorylated MST1/2 and LATS1/2 changed, we indeed observed that the expression of both MST1/2 and LATS1/2 total protein levels increased/decreased, as well as that there was reduced/increased YAP total protein expression accompanied by ARSD overexpression or knocking down. It is hard to explain why total protein levels of MST1/2, LATS1/2, and YAP changed along with ASRD overexpression or knocking down, which deserve to be addressed in future studies.

So far, to our knowledge, this is the first report on the expression regulation of ARSD by transcription factor, FOXA1, GATA3, and ERα through chromatin loop formation. TNBC exhibits the decreased ARSD expression. Its overexpression can inhibit the proliferation and migration of TNBC cells through activating Hippo/YAP pathway. TNBC is generally characterized by a poor prognosis and high rates of proliferation and metastases. Due to these aggressive features and lack of targeted therapies, we hope to exploit viable molecular targets for TNBC. ARSD should be the most promising potential therapeutic target. Next, the more profound experimental researches will be performed.

## Material and methods

### Patients and tissue samples

Human breast cancer specimens were collected from 102 patients who underwent surgical resections between 2015 and 2019 in the Cancer Hospital of Shantou University Medical College (Shantou, Guangdong Province, China). The specimens were immediately snap-frozen and kept at −80 °C until use. All subjects received consent and written informed consent was obtained from each patient. This study was approved by the Ethics Committee of Cancer Hospital of Shantou University Medical College and was performed in accordance with the Code of Ethics of World Medical Association (Declaration of Helsinki).

### Cell lines and cell culture

Human embryonic kidney cells line HEK-293T and Human breast cancer cell lines MDA-MB-231, MCF-7, BT549, T47D, SKBR3 were purchased from the Committee on Type Culture Collection of the Chinese Academy of Science (Shanghai, China). Cells were routinely cultured in Dulbecco modified Eagle medium (Gibco, USA) supplemented with 10% fetal bovine serum and 1% penicillin/streptomycin under an atmosphere of 5% CO_2_ at 37°C. Changing culture medium and passaging cells was performed according to the standard cell culture techniques to ensure cellular integrity.

### Immunohistochemical and immunocytochemical staining

5-μm sections were deparaffinized in xylene and rehydrated in graded alcohol series and ultrapure water. The sections were boiled in citrate buffer (pH 6.0) for antigen retrieval and immersed in 0.3% H_2_O_2_ for 10 min to block endogenous peroxidase activity. After blocking, the sections were incubated within ARSD primary antibody (1:200 dilution; Invitrogen, USA) overnight at 4 °C. Immunoreactivity was detected by DAB Substrate Kit (CST, USA). MCF-7 and MDA-MB-231 cells seeded on glass cover slips were fixed with 10% formaldehyde and stained as previously described. Immunostaining results were evaluated under a Leica microscope (400× magnification) by two experienced pathologists blind to each patient’s clinical information.

### Cell transfection

For overexpression of ARSD, HEK-293T or MDA-MB-231 cells were seeded into 100 mm culture dishes (BIOFIL, China) and incubated until 70% confluency. Plasmid pEX-C0483-M03/ARSD or respective empty vector pEX-NEG-M03 was encapsulated within the liposomes and transfected into cells by using Lipofectamine 3000 (ThermoFisher, USA). For ARSD or Xist RNA knockdown experiment, MCF-7 or MDA-MB-231 cells were cultured in 60 mm dishes and transfected with ARSD siRNA, Xist siRNA or control non-targeting siRNA. The transfected cells were incubated for 48–96 h.

### RNA isolation and quantitative real-time PCR

Total RNA was extracted from cell lines using TRIzol Reagent (Ambion, USA). RNA degradation and purification were performed according to the manufacturer’s instructions. The concentration and quality were determined by Nano Drop instrument (ThermoFisher, USA). For qRT-PCR experiments, complementary DNA (cDNA) was synthesized by using a PrimeScript RT reagent Kit (Takara, Japan) and amplified by using a SYBR qRT-PCR SuperMix Plus (Novoprotein, China). All primers were listed in Supplementary Table 1. The relative expression levels were normalized to GAPDH in this study. Each qRT-PCR was performed in triplicate.

### Protein extraction and Western blotting

To detect protein expression levels, cells were collected and lysed in ice-cold RIPA Lysis buffer (Beyotime, China). Equal amount of proteins (35 mg per lane) were separated in the 10% sodium dodecyl sulfate-polyacrylamide gel and transferred to a polyvinylidene fluoride membrane. The primary antibodies used in this study were listed in Supplementary Table 2. GAPDH was used as control to normalize the loading difference.

### Cell proliferative assay

The transfected cells were placed at a concentration of 500 cells/ml into 96-well culture plates. The replications were done with five wells plated same cells. The plates were incubated for 7 days. 10 µl of CCK-8 solution (BOSTER, USA) was added to each well every 24 h and the plates were incubated for 2 h. Absorbance was measured at 450 nm using a microplate reader (SpectraMax M5, Sunnyvale, CA, USA).

### Colony formation assay

Transfected cells at a density of 500 per well were reseeded in a 6‐well plate. After incubating for 14 days, the cells were fixed with 4% paraformaldehyde for 60 min and stained with 500ul of 1% Gentian Violet for 15 min, followed by washing with phosphate-buffered saline (PBS). The numbers of colonies were counted under the photographic stereoscopic microscope. All experiments were repeated in triplicate.

### Wound healing assay

Wound healing assay was performed for analysis of cell migration in vitro. After transfecting with ARSD and empty vector, the cells were incubated for 72 h until 90% confluency. The cell monolayer was scratched by using 1ul pipette tip to create a wound and washed in PBS for three times. The cells were still incubated in DMEM containing with 10% fetal bovine serum and 1% penicillin/streptomycin for 60 h. Five random widths of the wound region were measured under a microscope (100× magnification) every 12 h.

### Transwell migration assay

The transwell migration assays were performed using transwell chambers (8‐μm pore size; Falcon) without Matrigel. Transfected cells were reseeded in the upper insert of the chambers with serum-free medium. Complete medium was added in the bottom chambers. After 72 h of incubation, the cells were fixed with 4% paraformaldehyde and stained with 1% Gentian Violet. Only the cells that had migrated to the lower surface of the membrane were counted under the microscope (scale bar = 200 μm). All assays were performed in triplicate.

### Demethylation treatment, DNA extraction, and methylation-specific PCR

To study the epigenetic modification of ARSD, genomic DNA of MCF-7 and MDA-MB-231 cells was extracted by using TIANamp Genomic DNA Kit (TIANGEN, China). Methyl Primer Express Software v. 1.0 was used to analyze the ARSD gene promoter and to design Methylation-specific PCR (MSP) primers (Supplementary Table 1). After DNA extraction and purification, MSP Kit (TIANGEN, China) was used to analyze the methylation characteristics of ARSD. To determine the methylation status of ARSD promoter region, MDA-MB-231 cells were treated with 5-Aza or RG108 for 5 days, while cells treated with DMSO were used as control group. The following steps were performed as described above.

### Luciferase reporter gene assay

Transient transfection with luciferase reporter constructs was performed using Lipofectamine 3000 in 6-well plates as previously described. Established FOXA1, GATA3 or ERα overexpressing HEK-293T cells and control HEK-293T cells were co-transfected with Renilla luciferase reporter vector and pGL3-basic-ARSD-promoter reporter vector, pGL3-basic vector or pGL3-control vector. Two days after transfection, firefly and Renilla luciferase levels were determined by luminometer using the Dual-Luciferase Reporter Assay Kit (Promega, USA) according to the manufacturer’s instructions. All experiments were repeated in triplicate.

### Chromatin immunoprecipitation (ChIP) and ChIP-qPCR

To determine whether the FOXA1, GATA3, and ERα regulate the expression of ARSD, ChIP was performed using ChIP Assay Kit (Beyotime, China) following the manufacturer’s instructions. Briefly, MCF-7 cells were fixed with 1% final concentration of formaldehyde and terminated by glycine solution at room temperature. Cross-linked chromatin was sheared by sonication using a E220 Focused-Ultrasonicator (Covaris, USA). The supernatant was incubated with primary antibody anti-FOXA1, anti-GATA3, anti-ERα or a negative IgG antibody overnight at 4 °C. Chromatin-antibody complexes were deposited using protein A+G agarose resin and resuspended using washing buffer. After purifying, immunoprecipitated DNA was analyzed by semi-quantitative PCR. ChIP primer sequences were provided in Supplementary Table 1. To determine whether EZH2, 5-Methylcytosine (5-mC), H3K27me1, H3K27me2, and H3K27me3 occupy on the ARSD promoter, ChIP-qPCR was performed as previously described with minor modifications.

### Chromosome conformation capture (3C)

MDA-MB-231 and MCF-7 cells were grown in 100-mm dishes containing DMEM supplemented with 10% FBS and 1% penicillin/streptomycin. Before harvesting, DNA was cross-linked by 1% final concentration of formaldehyde at 37 °C for 10 min and terminated by glycine solution at room temperature for 5 min. Cells were scraped from dishes, lysed in cold SDS Lysis Buffer containing 1 mM PMSF, and disrupted by Dounce Homogenizer on ice. After centrifugation, the nuclei were resuspended in 1× NEB buffer and digested by restriction enzymes HpaI and AfeI at 37 °C for 3 h. The digested products were incubated overnight with T4 DNA ligase at 16 °C. DNA cross-links were added with proteinase K and NaCl and incubated overnight at 65 °C. After DNA extraction, the 3 C products were analyzed by semi-quantitative PCR and qRT-PCR.

### Statistical analysis

Statistical analysis was performed using GraphPad prism 6 software. Differences between the individual means were determined using Student’s *t*-test. The correlation between ARSD immunohistochemical staining and the clinicopathologic characteristics of breast cancer patients was determined by Chi-square test. *p* < 0.05 was considered as statistically significant difference.

### Abbreviations used in this article

BC breast cancer, LN lymph node, Top IIα Topoisomerase II alpha, ChIP chromatin immunoprecipitation, CHIP-3C chromosome conformation capture, ARSD arylsulfatase D, XCI X chromosome inactivation, PR+ progesterone receptor positive, ER+ estrogen receptor positive, 5-Aza 5′-Azadeoxycytidine.

RG108 a non-nucleoside DNA methyltransferase inhibitor, *** for *p* < 0.001; ** for *p* < 0.01; * for *p* < 0.05.

## Materials availability

The datasets used and/or analyzed during the current study are available from the corresponding author on reasonable request.

## Supplementary information

Supplementary legend

Supplementary Figure 1

Supplementary Figure 2

Supplementary Figure 3

Supplementary Figure 4

Supplementary Figure 5

Supplementary Figure 6

Supplementary Figure 7

SupplementayFigure 8

Supplementary Table 1

Supplementary Table 2

## Data Availability

The authors declare that the data are transparent.
