# Supplementary material for: ARSD, a novel ERα downstream target gene, inhibits proliferation and migration of breast cancer cells via activating Hippo/YAP pathway
Source: Cell Death Dis. 2021 Nov 2;12(11):1042. doi: 10.1038/s41419-021-04338-8 (PMC8560752; doi:10.1038/s41419-021-04338-8)
Supplement: Supplementary file 1 — Supplementary legend [file 41419_2021_4338_MOESM1_ESM.docx]

**Supplement figure legend：**

**Supplementary Figure 1. The mRNA expression of second family members of arylsulfatase genes mapped to Xp22.3, i.e., SDS, ARSD, ARSE. ARSF, ARSH, in different tumor types.**

**(A)** The data on transcript expression for STS, ARSD, ARSE. ARSF, ARSH, are extracted from the database Oncomine (<https://www.oncomine.org/>). The number of Oncomine profiles with significant gene over-expression (red) or under-expression (blue) for each combination (tissue-type and analysis-type) is recorded in the appropriate box. Colors indicate direction and the color intensity equals the percentile, i.e., Top 1%, 5%, or 10% significantly over- or under-expressed. The datasets were obtained with the following parameters: p value threshold of 0.01. ARSD expression is decreased in breast cancer in comparison to the normal tissue.

**Supplementary Figure 2**

**(A)** Correlation analysis between ARSD expression level and clinicopathologic characteristics, including BC subtype, age, p53 status, BRAC1/2 status and Ki67 positive level by using Breast Cancer Gene-Expression Miner v4.6 online tool. ARSD presents lower expression in basal like breast cancer and TNBC; in ≤51; in mutated p53 status; in mutated BRAC1/2 cases; in Ki67 high cases. **(B)** Full membranes of western blot assays in Figure 2D. **(C)** Full membranes of western blot assays in Figure 2F.

**Supplementary Figure 3:** Wound healing assay using mouse TNBC 4T1 cells.

**Supplementary Figure 4:**

**(A)** Wound healing assay of BT-549/NC cells and BT-549/ARSD cells. **(B)** Colony formation of BT-549/NC cells and BT-549/ARSD cells on a plastic substrate. The colony formation rate was quantified using ImageJ software 14 days after plating.  **(C)** Cell proliferation analysis of negative control (BT-549/NC cells) and BT-549/ARSD cells is detected every 24h after plating (day 0) by using CCK-8 assay. **(D)** Transwell assay of BT-549/NC cells and BT-549/ARSD cells. **(E)** Cell proliferation analysis of negative control (MCF-7/siNC cells) and MCF-7/siARSD cells is detected every 24h after plating (day 0) by using CCK-8 assay. **(F)** Transwell assay of MCF-7/siNC cells and MCF-7/siARSD cells. **(G)** Wound healing assay of MCF-7/siNC cells and MCF-7/siARSD cells. **(H)** Colony formation of MCF-7/siNC cells and MCF-7/siARSD cells on a plastic substrate. The colony formation rate was quantified using ImageJ software 14 days after plating; Error bars are ±SEM (* p < 0.05, ** p < 0.01, *** p < 0.001).

**Supplementary Figure 5:** Correlation analyses between ARSD and Luminal subtype transcriptional factors, e.g., FOXA1 **(a)**, GATA3 **(b)**, ESR1 **(c)** and SPDEF **(d)**, exhibit higher expression in luminal subtype breast cancer, and lower expression in TNBC by using GEPIA online tool.

**Supplementary Figure 6:** Full membranes of western blot assays in Figure 4D and 4E.

**Supplementary Figure 7:** For 3C experiment, analyzing restriction enzyme sites within ARSD enhancer/promoter and choosing restriction enzyme.

**Supplementary Figure 8: Correlation analyses between ARSD and major signal pathways.**

There is a strong correlation between ARSD and Hippo/YAP signaling pathway when compared with other signaling pathways.

**Supplement table legend：**

**Supplement table 1: Primers and small fragment sequences used in this study**

**Supplement table 2: Antibodies used in this study**
