# Supplementary figures and images for: ARSD, a novel ERα downstream target gene, inhibits proliferation and migration of breast cancer cells via activating Hippo/YAP pathway
Source: Cell Death Dis. 2021 Nov 2;12(11):1042. doi: 10.1038/s41419-021-04338-8 (PMC8560752; doi:10.1038/s41419-021-04338-8)

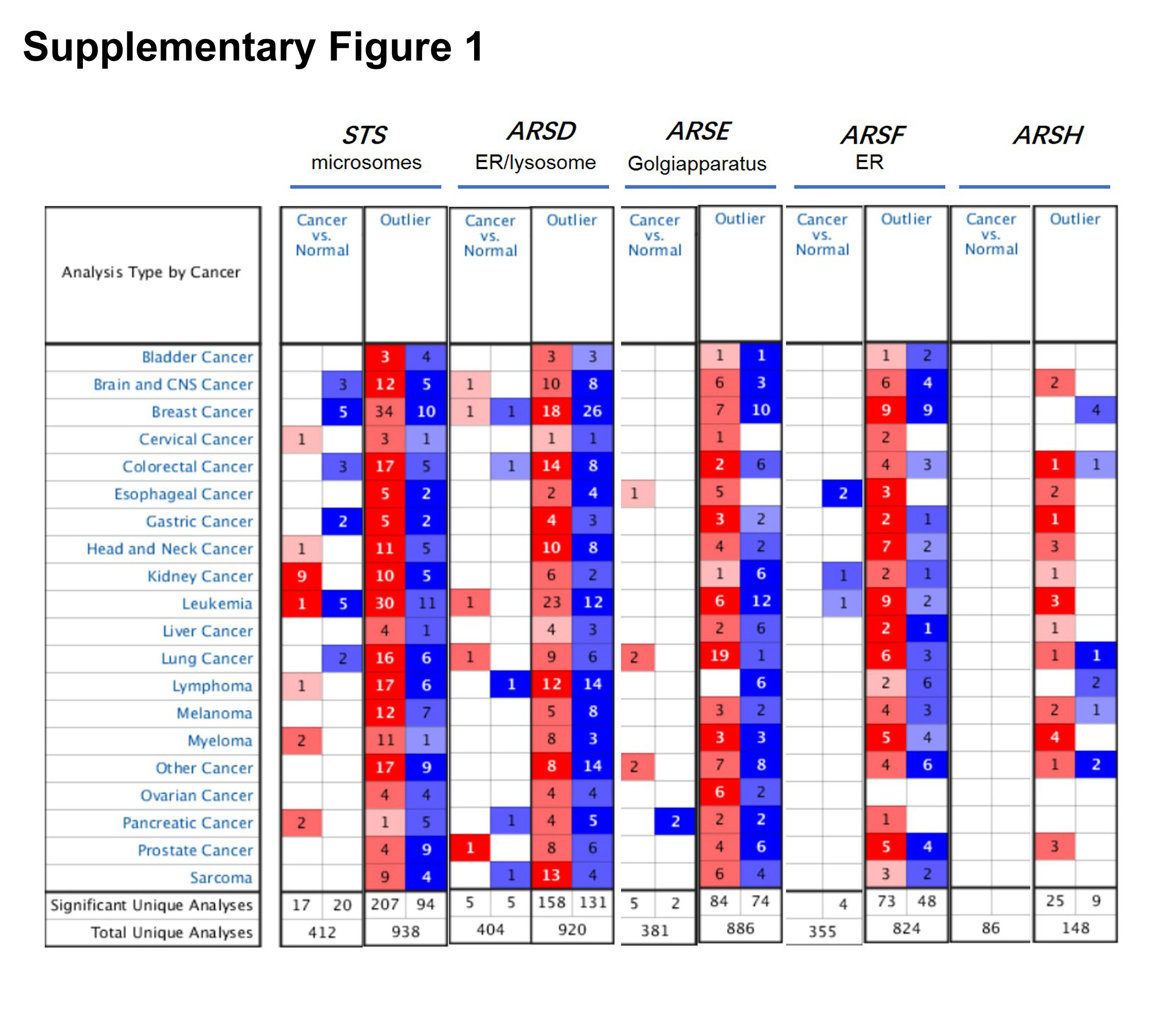

Supplement: Supplementary file 2 — Supplementary Figure 1 [file 41419_2021_4338_MOESM2_ESM.jpg]

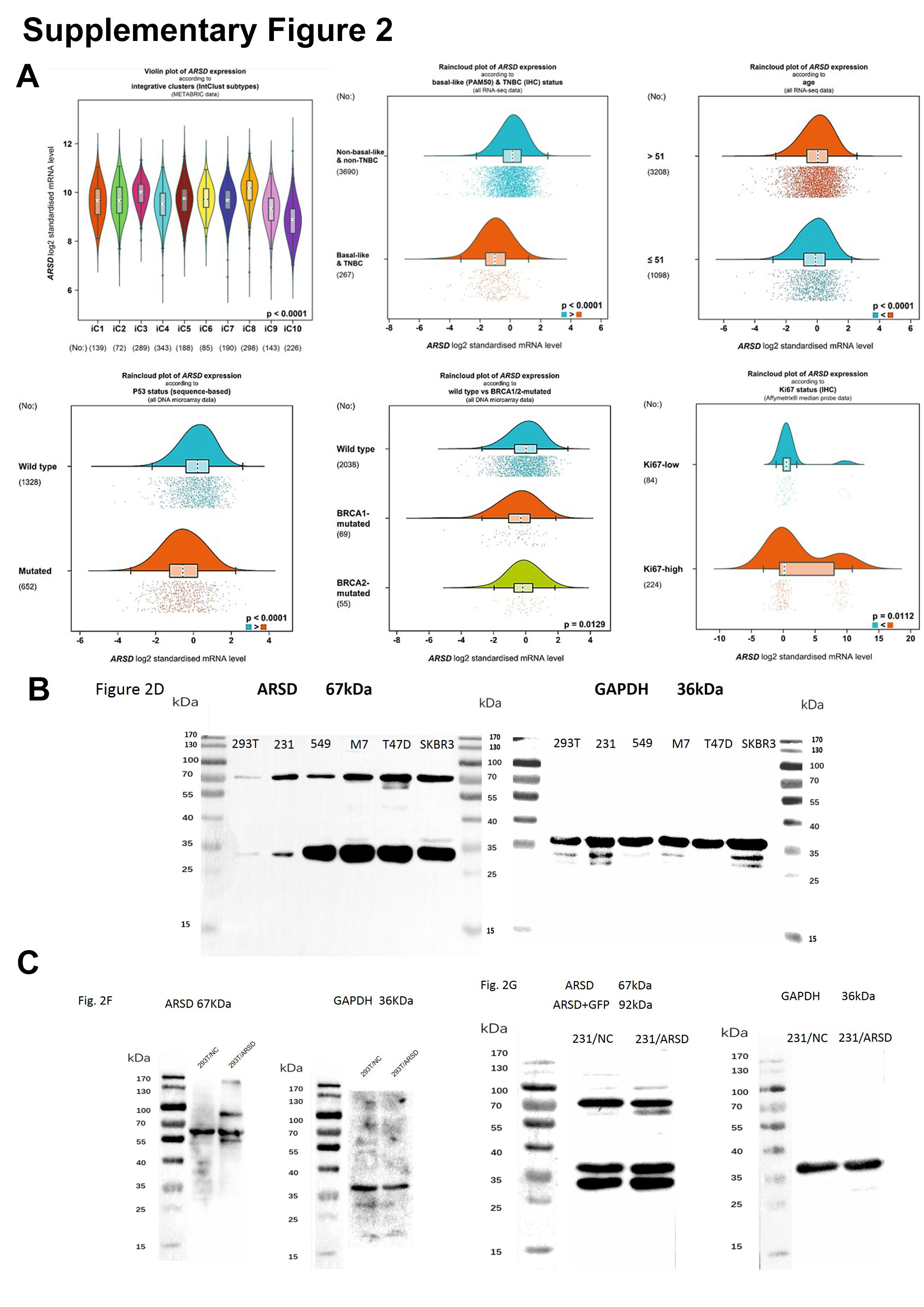

Supplement: Supplementary file 3 — Supplementary Figure 2 [file 41419_2021_4338_MOESM3_ESM.jpg]

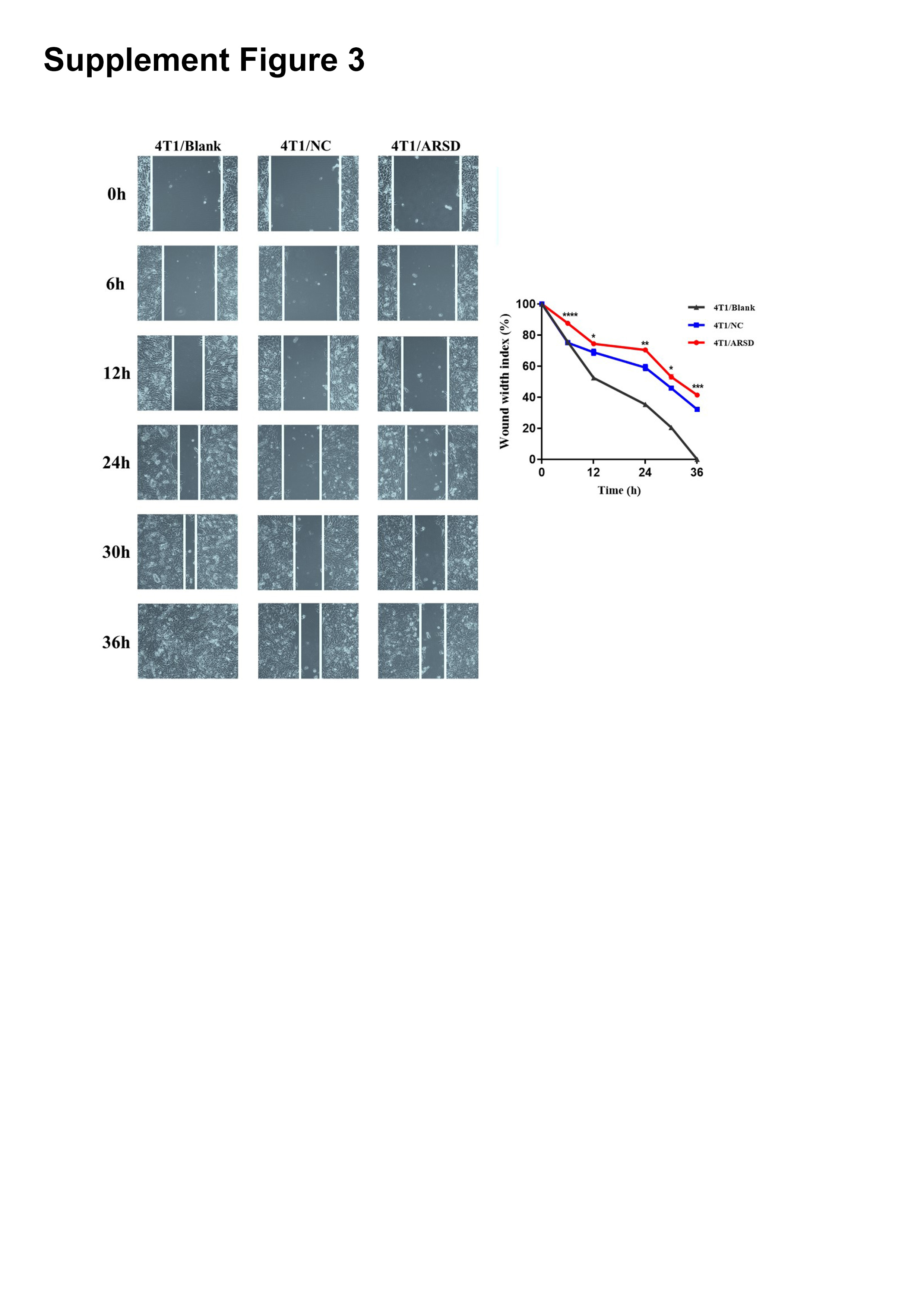

Supplement: Supplementary file 4 — Supplementary Figure 3 [file 41419_2021_4338_MOESM4_ESM.jpg]

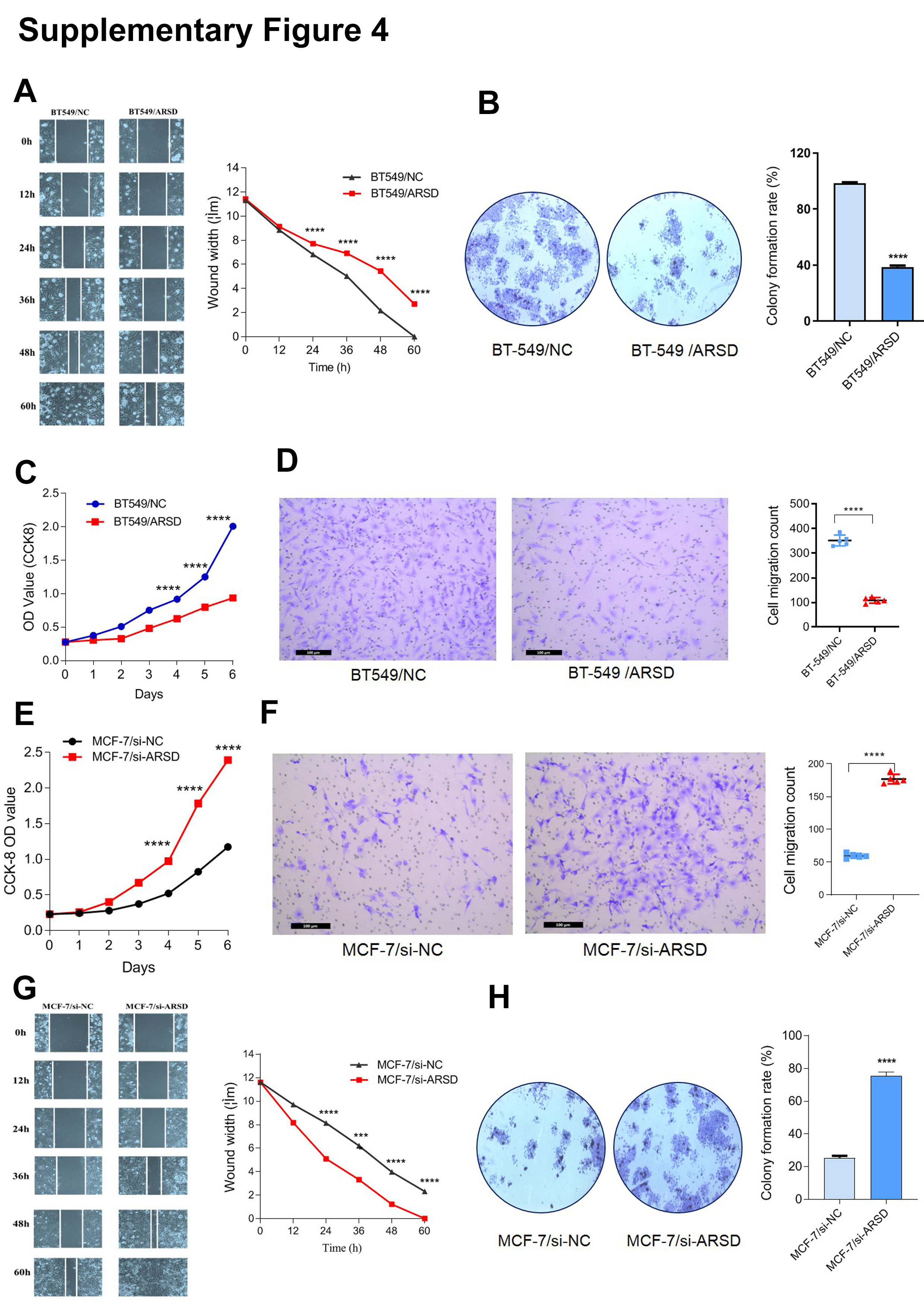

Supplement: Supplementary file 5 — Supplementary Figure 4 [file 41419_2021_4338_MOESM5_ESM.jpg]

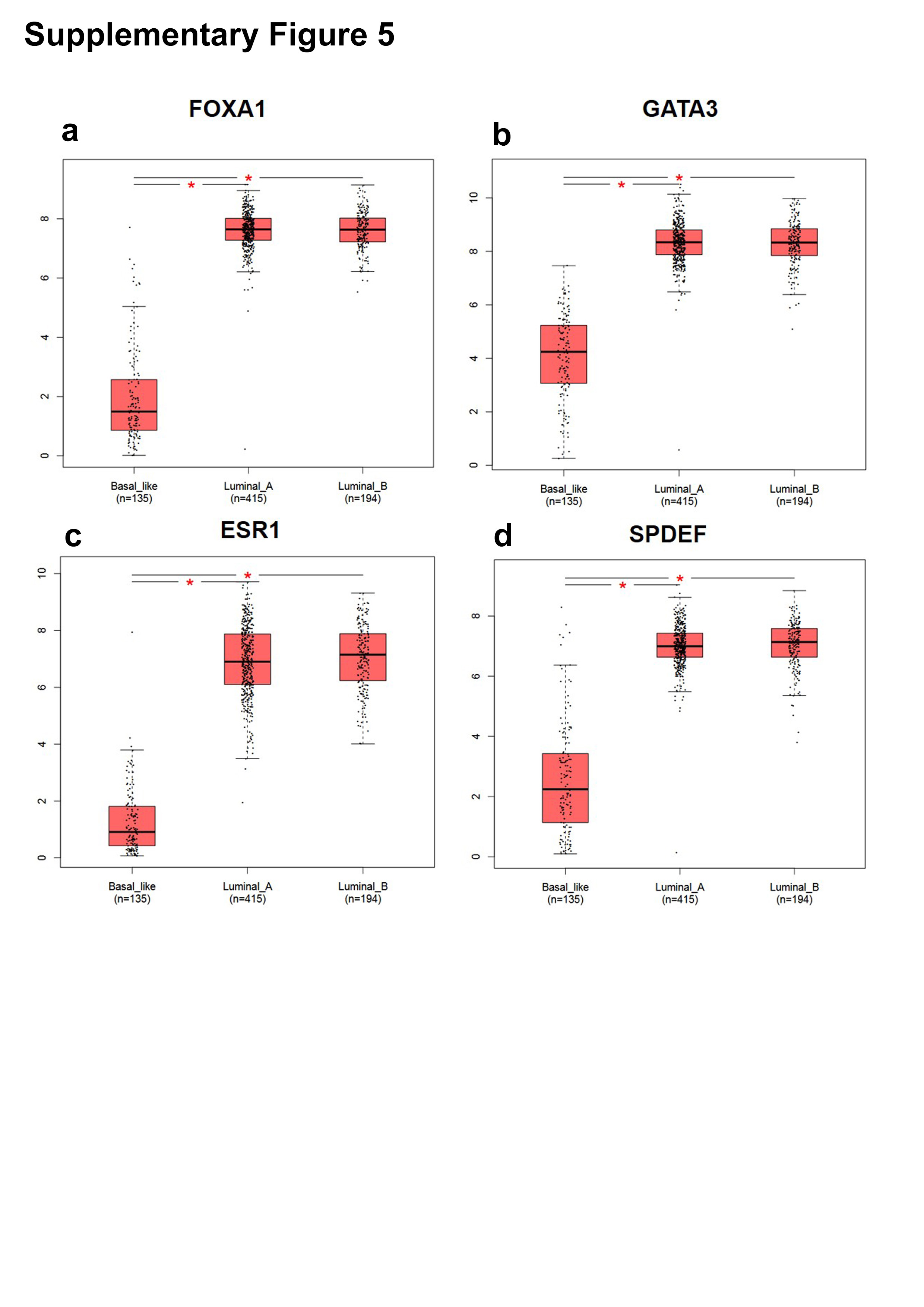

Supplement: Supplementary file 6 — Supplementary Figure 5 [file 41419_2021_4338_MOESM6_ESM.jpg]

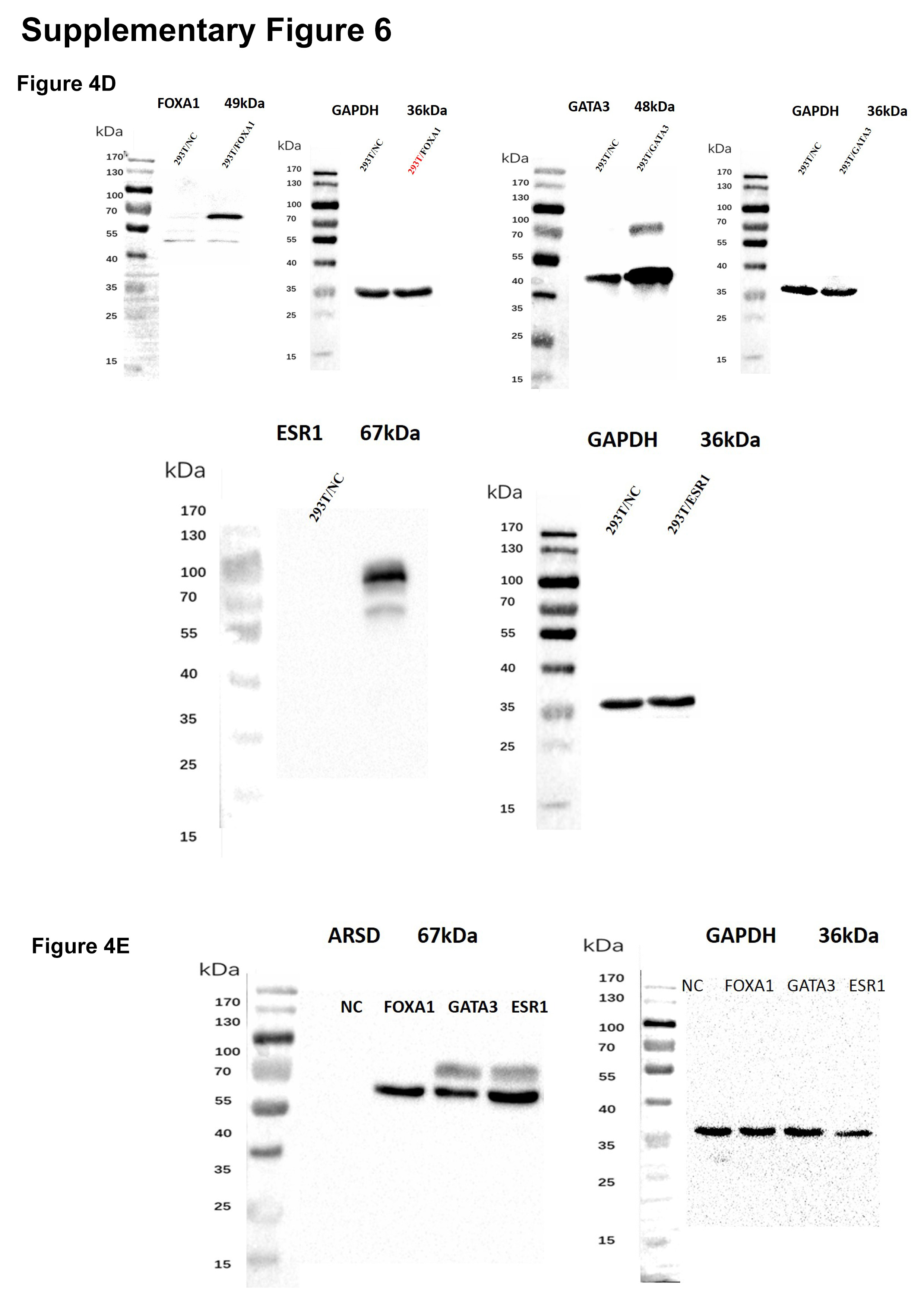

Supplement: Supplementary file 7 — Supplementary Figure 6 [file 41419_2021_4338_MOESM7_ESM.jpg]

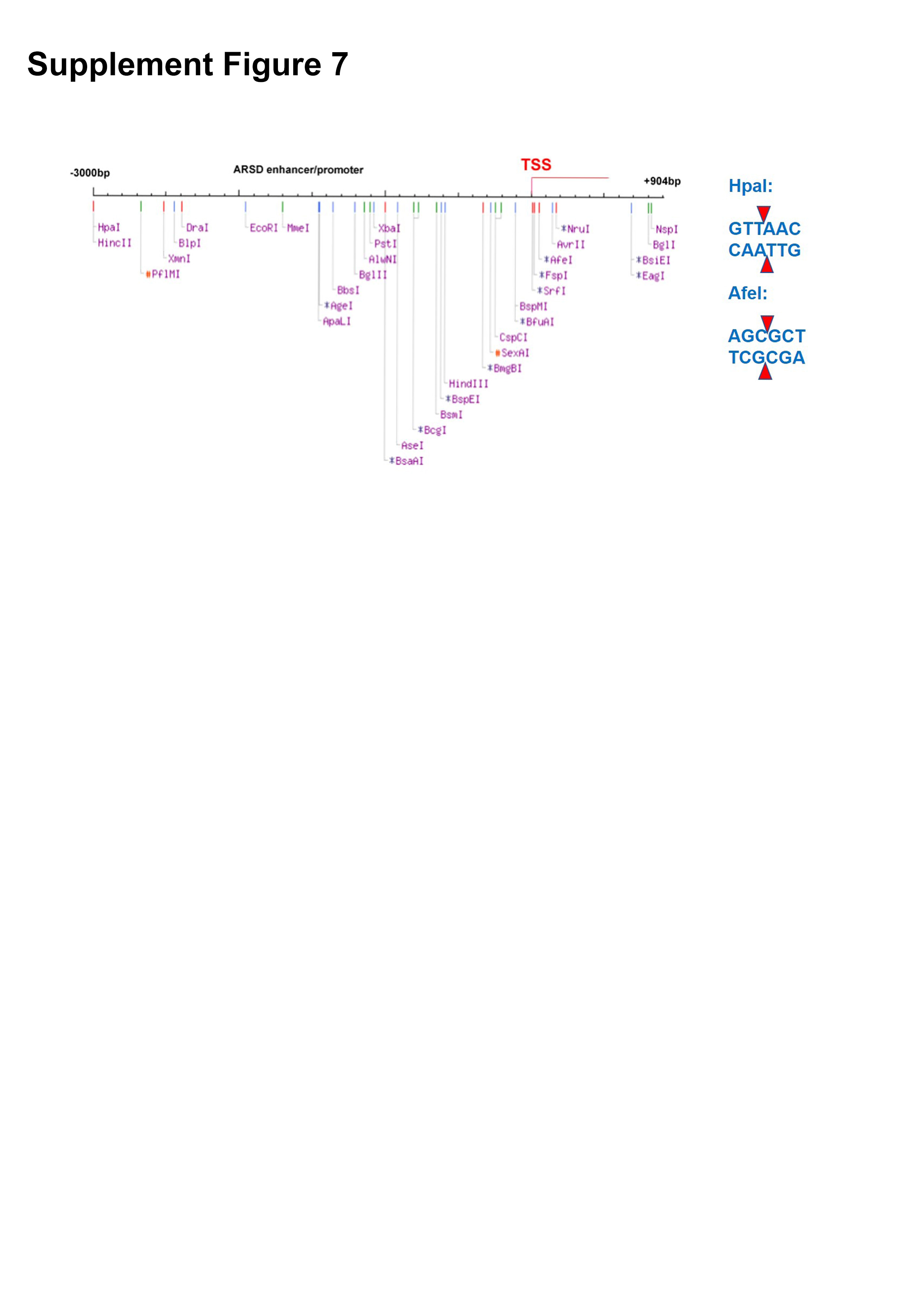

Supplement: Supplementary file 8 — Supplementary Figure 7 [file 41419_2021_4338_MOESM8_ESM.jpg]

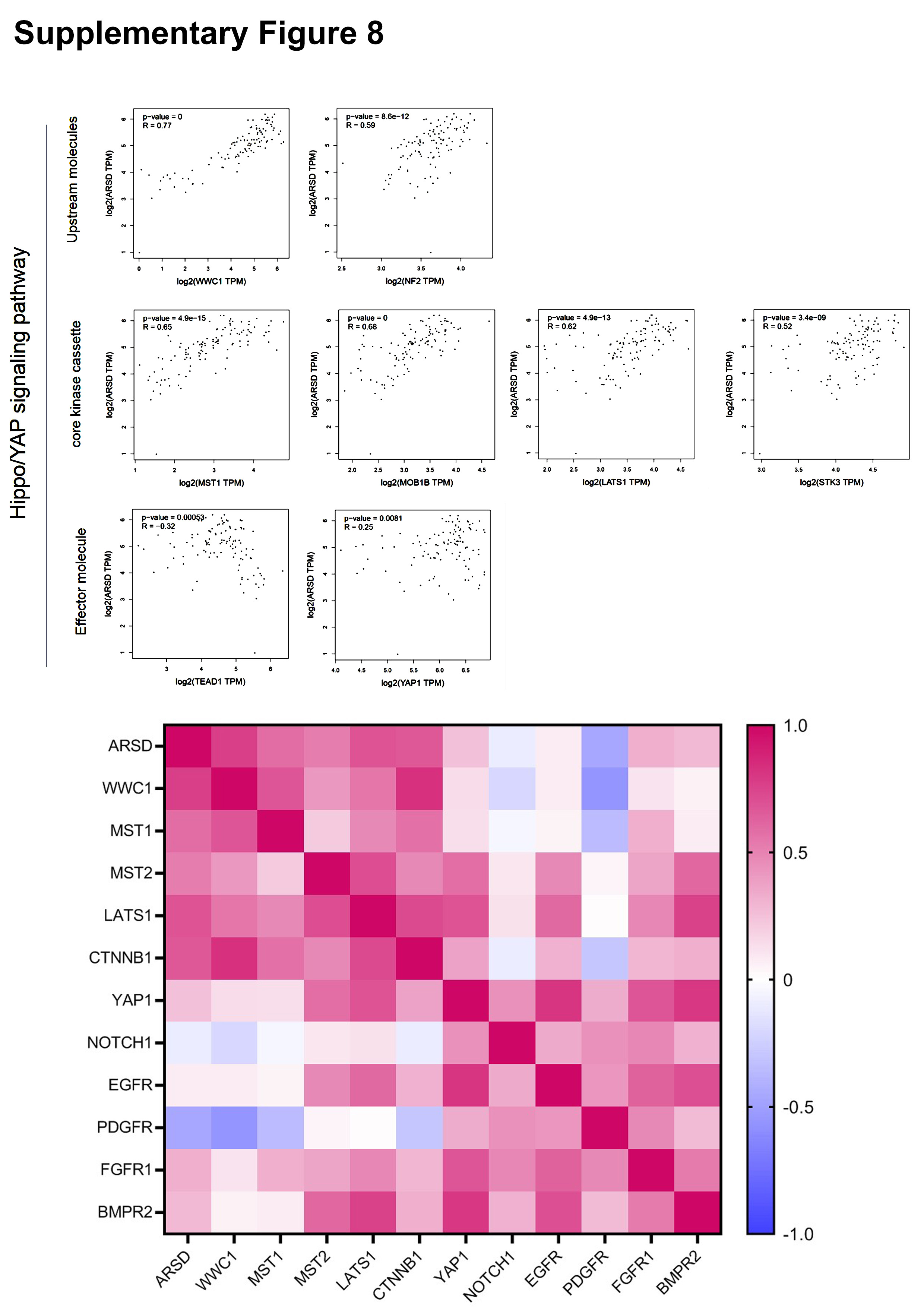

Supplement: Supplementary file 9 — SupplementayFigure 8 [file 41419_2021_4338_MOESM9_ESM.jpg]
