# Supplementary material for: ARSD, a novel ERα downstream target gene, inhibits proliferation and migration of breast cancer cells via activating Hippo/YAP pathway
Source: Cell Death Dis. 2021 Nov 2;12(11):1042. doi: 10.1038/s41419-021-04338-8 (PMC8560752; doi:10.1038/s41419-021-04338-8)
Supplement: Supplementary file 10 — Supplementary Table 1 [file 41419_2021_4338_MOESM10_ESM.docx]

Table1: Primers and small fragment sequences used in this study

| Gene | Forward primer | Reverse primer | Size | Aim |
| --- | --- | --- | --- | --- |
| ARSD | CGAGCTCTGTGCTGAAAGTCATGTGAGA | TCCCCCGGGCTTCTCTCTTTGCCCTGTTACT | 750bp | promoter part1 |
| ARSD | CGAGCTCCTCAACTCTCAGCCTTGATCTT | TCCCCCGGGCTTCCCTTCCTCCTTTGTTCTC | 1503bp | Promoter part2 |
| ARSD | AGGGTGTGAATTGTGCATCCC | ACAGTCGTTTGTGAGCGTGAA | 101bp | RT-PCR |
| ARSD | GAGACCAGCTTAGGCAACATAG | CAGGTCCTCCTCACCATTATTC | 1580bp | CHIP-3C |
| ARSD | CGAGCTCTGTGCTGAAAGTCATGTGAGA | TCCCCCGGGCTTCTCTCTTTGCCCTGTTACT | 750bp | Luciferase assay |
| ARSD | GGATTTTAGGCGGGAGTTATC | CCTAACTCTCGAAACCTCCG |  | MSP (M) |
| ARSD | TGGGATTTTAGGTGGGAGTTATT | CCTAACTCTCAAAACCTCCAAA |  | MSP (UM) |
| ARSD | TGTGCTGAAAGTCATGTGAGA | CTTCTCTCTTTGCCCTGTTACT | 180bp | CHIP-PCR |
| Xist | GTGATCAGCACCCCAGCTAT | TCTTTCTTTTCCTCCCAGCA | 192bp | RT-PCR |
| FOXA1 | GCAATACTCGCCTTACGGCT | TACACACCTTGGTAGTACGCC | 120bp | RT-PCR |
| GATA3 | GCCCCTCATTAAGCCCAAG | TTGTGGTGGTCTGACAGTTCG | 80bp | RT-PCR |
| ESR1 | CCCACTCAACAGCGTGTCTC | CGTCGATTATCTGAATTTGGCCT | 180bp | RT-PCR |
| GAPDH | TATGTCGTGGAGTCTACTGGT | GAGTTGTCATATTTCTCGTGG |  | RT-PCR |
| siXist-#1 | GCUGACUACCUGAGAUUUATT | UAAAUCUCAGGUAGUCAGCTT |  | Knocking down |
| siXist-#2 | GCAUGCAUCUUGGACAUUUTT | AAAUGUCCAAGAUGCAUGCTT |  | Knocking down |
| SiARSD-#1 | GGGCUGUGAUGAUUCUUCUTT | AGAAGAAUCAUCACAGCCCTT |  | Knocking down |
| SiARSD-#2 | GGGACUGCUUCAAGAGAAATT | UUUCUCUUGAAGCAGUCCCTT |  | Knocking down |
