# Supplementary material for: ARSD, a novel ERα downstream target gene, inhibits proliferation and migration of breast cancer cells via activating Hippo/YAP pathway
Source: Cell Death Dis. 2021 Nov 2;12(11):1042. doi: 10.1038/s41419-021-04338-8 (PMC8560752; doi:10.1038/s41419-021-04338-8)
Supplement: Supplementary file 11 — Supplementary Table 2 [file 41419_2021_4338_MOESM11_ESM.docx]

Table 2: Antibodies used in this study

| Gene | Company | No. Clone | Catalog No. | Species | Con. | Purpose | Size (kDa) |
| --- | --- | --- | --- | --- | --- | --- | --- |
| ARSD | Invitrogen |  | PA5-52087 | Rabbit | WB 1:500  IHC 1:200 | WB, IHC | 65 |
| GFP Tag | proteintech |  | 50430-2-AP | Rabbit | 1:800 | WB | 26 |
| FOXA1 | abcam | EPR10881 | ab170933 | Rabbit | 1:1000 | WB, ChIP | 49 |
| GATA3 | abcam | EPR16651 | ab199428 | Rabbit | 1:1000 | WB, ChIP | 48 |
| ESR1 | abcam | E115 | ab32063 | Rabbit | 1:1000 | WB, ChIP | 67 |
| P-LATS1 | CST | Ser909 | #9157 | Rabbit | 1:1000 | WB | 140 |
| LATS2 | CST | D83D6 | #5888 | Rabbit | 1:1000 | WB | 150 |
| Merlin | CST | D3S3W | #12888 | Rabbit | 1:1000 | WB, IHC | 70 |
| KIBRA | CST |  | #8774 | Rabbit | 1:1000 | WB | 135 |
| P-MST1/MST2 | Invitrogen | Thr183 | PA5-40255 | Rabbit | 1:1000 | WB, IHC | 59 |
| YAP1 | abcam |  | ab76252 | Rabbit | 1:1000 | WB | 75 |
| LATS1 | Boster |  | A01051-2 | Rabbit | 1:1000 | WB | 127 |
| P-LATS2 | Affinity |  | AF7439 | Rabbit | 1:1000 | WB | 150 |
| MST1/MST2 | Affinity |  | DF8569 | Rabbit | 1:1000 | WB | 56 |
| P-YAP | Affinity |  | AF3328 | Rabbit | 1:1000 | WB | 78 |
| GAPDH | proteintech |  | 10494-1-AP | Rabbit | 1:10000 | WB,IHC | 36 |
